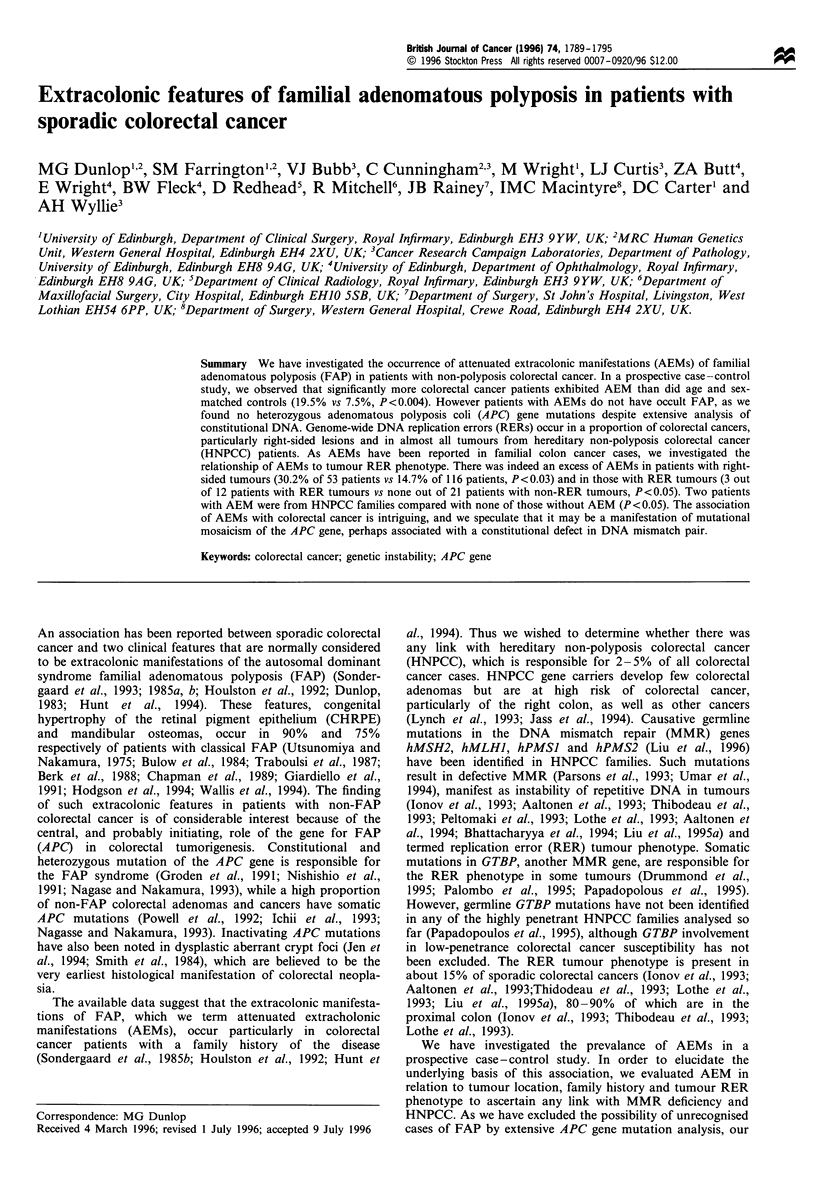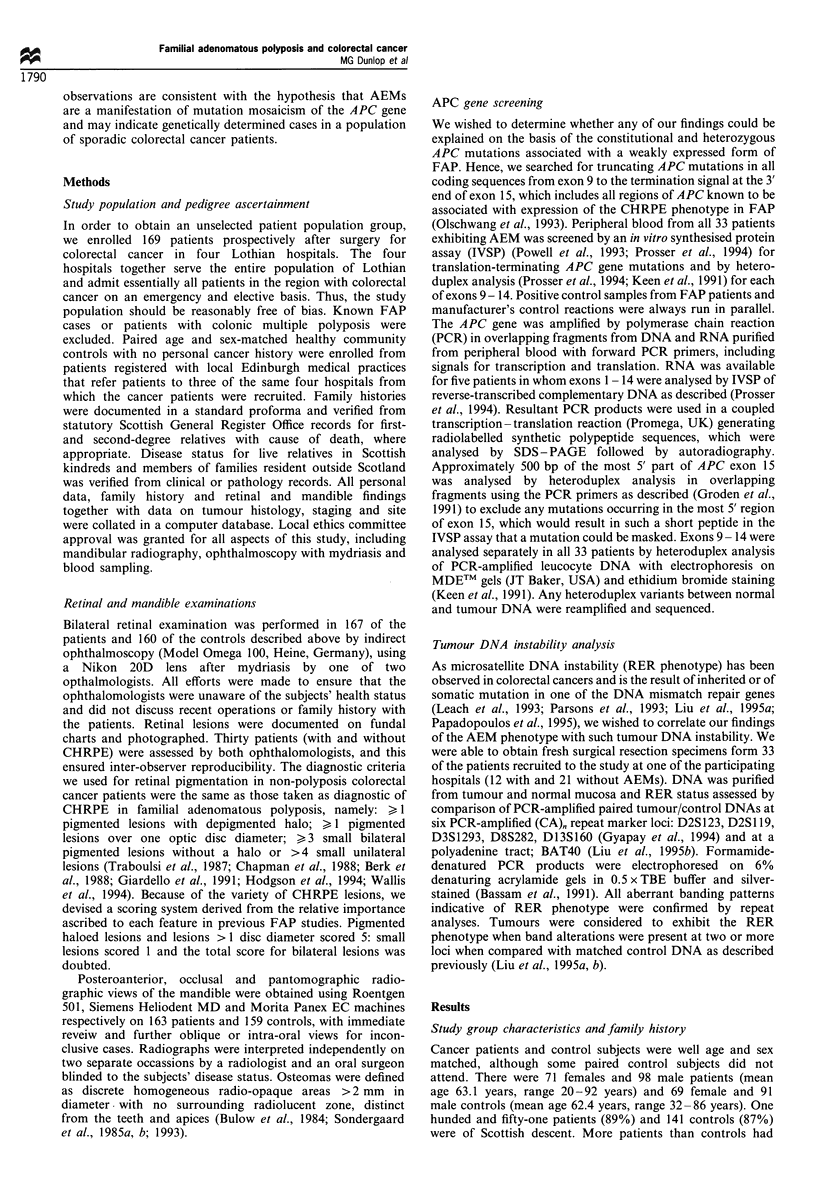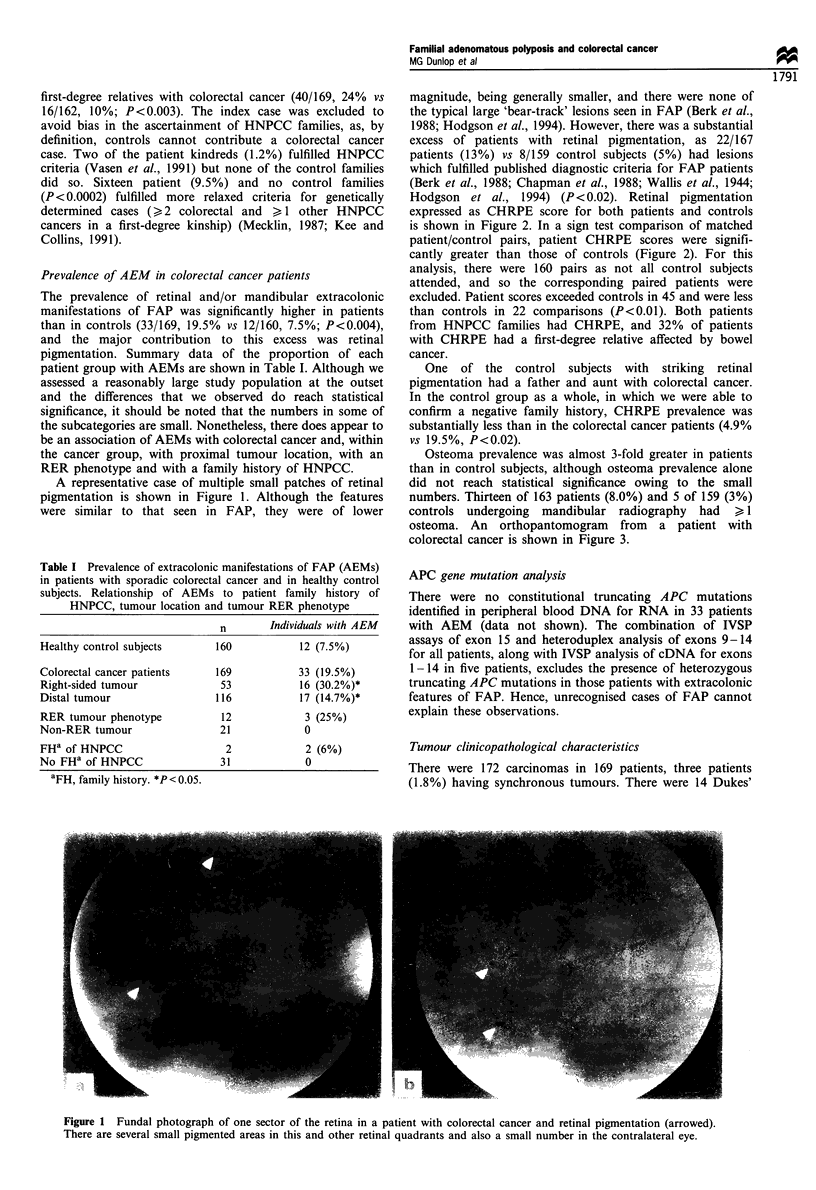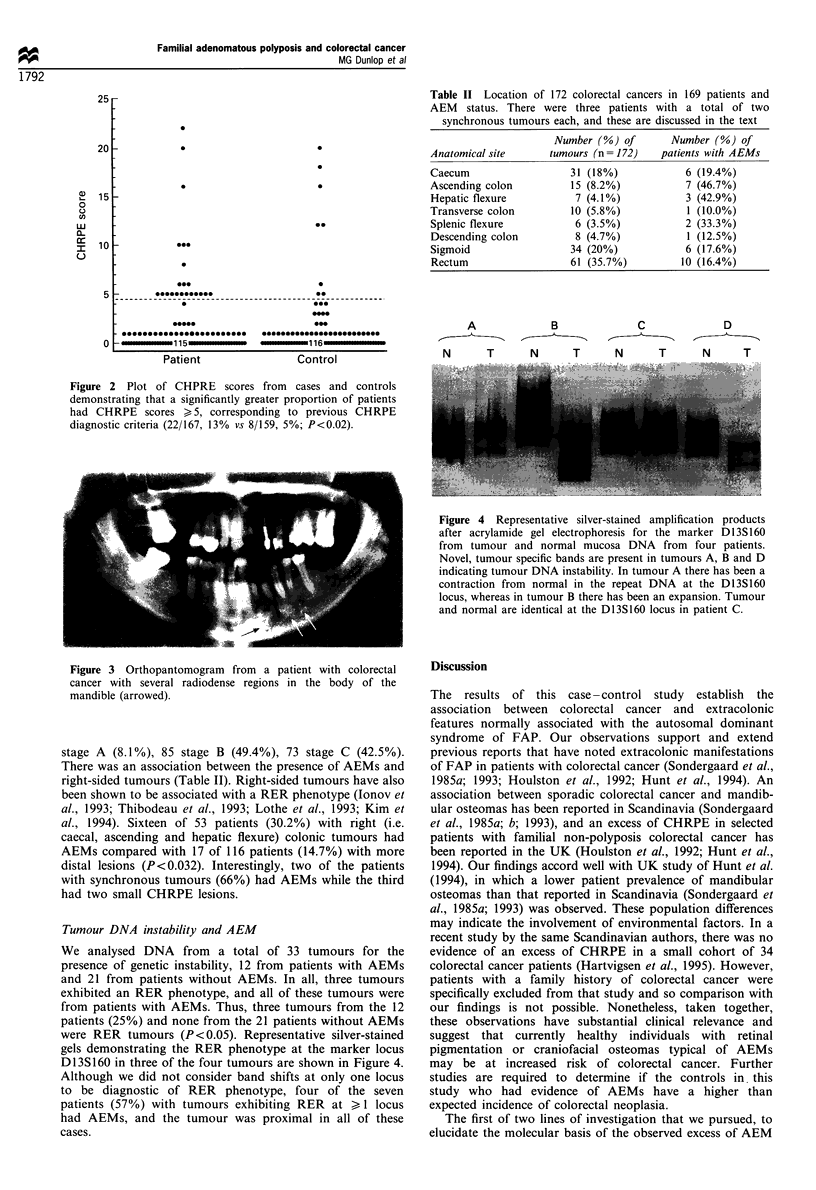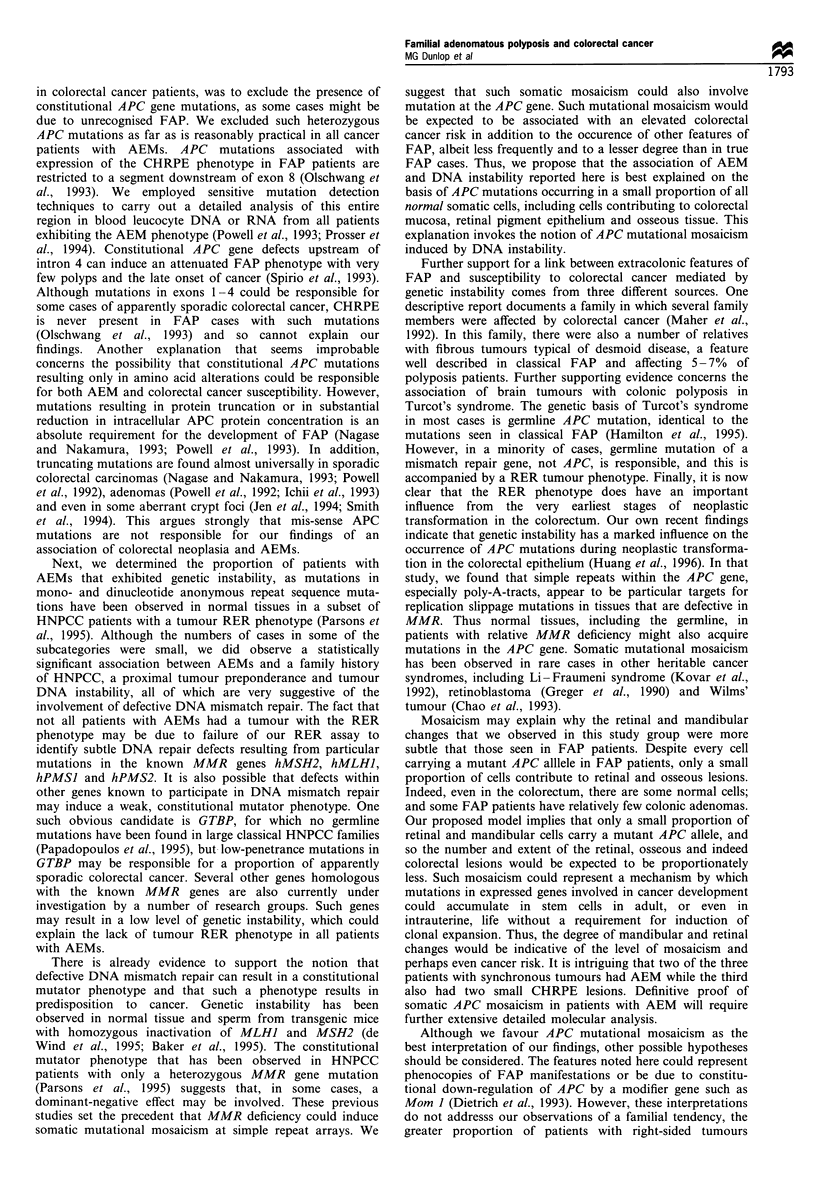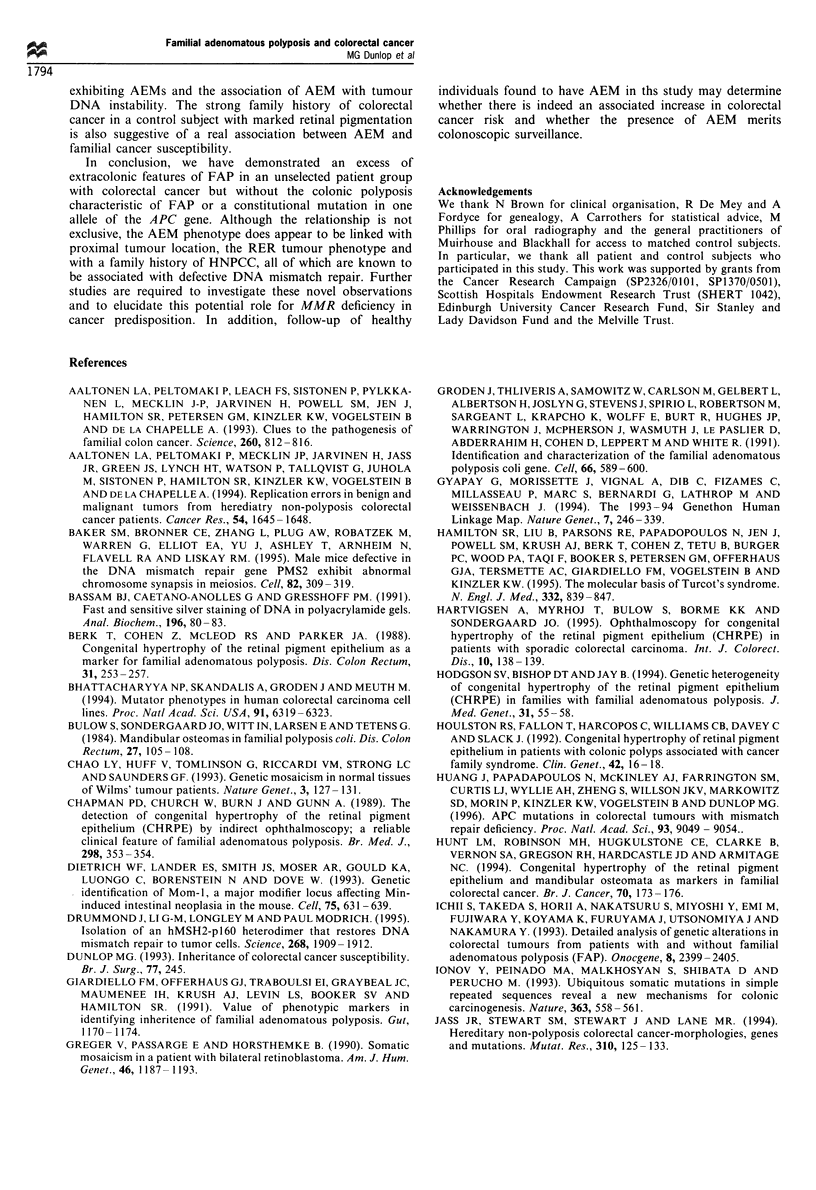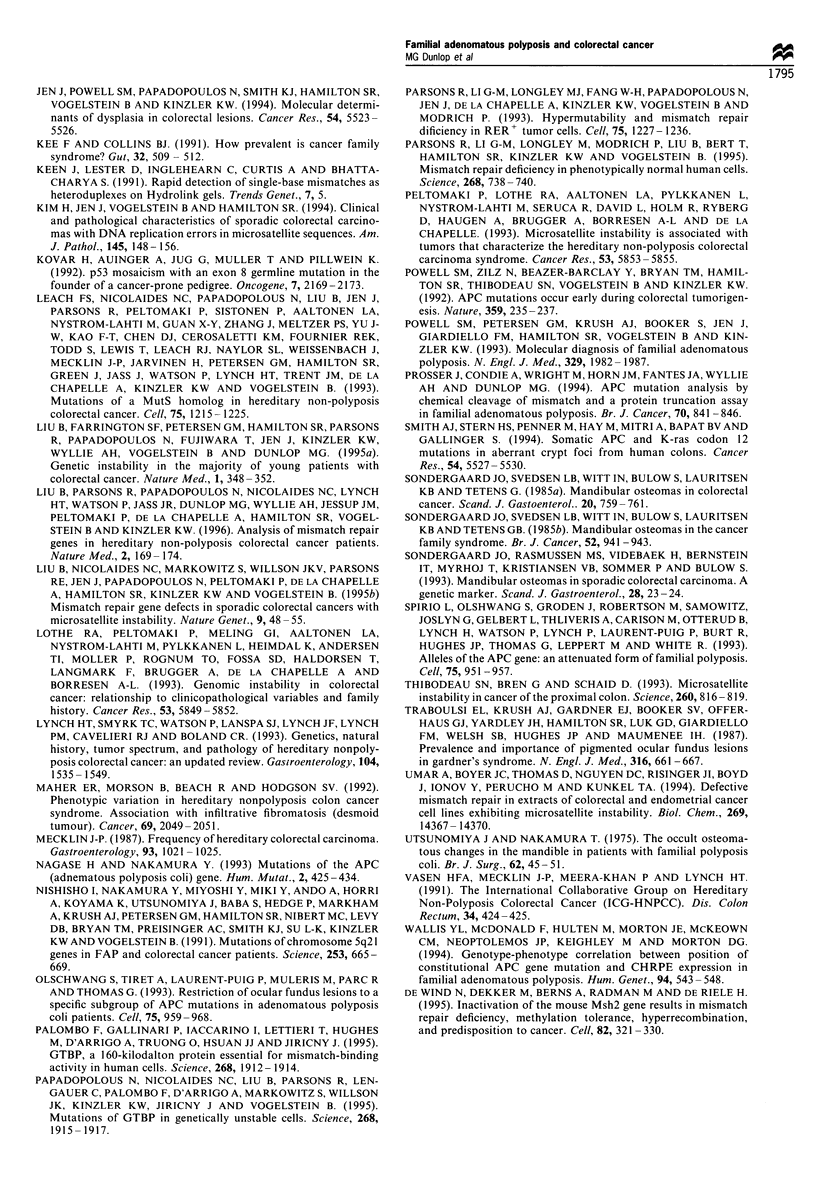# Extracolonic features of familial adenomatous polyposis in patients with sporadic colorectal cancer.

**DOI:** 10.1038/bjc.1996.631

**Published:** 1996-12

**Authors:** M. G. Dunlop, S. M. Farrington, V. J. Bubb, C. Cunningham, M. Wright, L. J. Curtis, Z. A. Butt, E. Wright, B. W. Fleck, D. Redhead, R. Mitchell, J. B. Rainey, I. M. Macintyre, D. C. Carter, A. H. Wyllie

**Affiliations:** University of Edinburgh, Department of Clinical Surgery, Royal Infirmary, UK.

## Abstract

**Images:**


British Journal of Cancer (1996) 74, 1789-1795
© 1996 Stockton Press All rights reserved 0007-0920/96 $12.00

### Extracolonic features of familial adenomatous polyposis in patients with sporadic colorectal cancer

MG Dunlop[1,2], SM Farrington[1,2], VJ Bubb[3], C Cunningham[2,3], M Wright[1], LJ Curtis[3], ZA Butt[4], E Wright[4], BW Fleck[4], D Redhead[5], R Mitchell[6], JB Rainey[7], IMC Macintyre[8], DC Carter[1] and AH Wyllie[3]

*[1]University of Edinburgh, Department of Clinical Surgery, Royal Infirmary, Edinburgh EH3 9YW, UK; [2]MRC Human Genetics Unit, Western General Hospital, Edinburgh EH4 2XU, UK; [3]Cancer Research Campaign Laboratories, Department of Pathology, University of Edinburgh, Edinburgh EH8 9AG, UK; [4]University of Edinburgh, Department of Ophthalmology, Royal Infirmary, Edinburgh EH8 9AG, UK; [5]Department of Clinical Radiology, Royal Infirmary, Edinburgh EH3 9YW, UK; [6]Department of Maxillofacial Surgery, City Hospital, Edinburgh EH10 5SB, UK; [7]Department of Surgery, St John's Hospital, Livingston, West Lothian EH54 6PP, UK; [8]Department of Surgery, Western General Hospital, Crewe Road, Edinburgh EH4 2XU, UK.*

**Summary** We have investigated the occurrence of attenuated extracolonic manifestations (AEMs) of familial adenomatous polyposis (FAP) in patients with non-polyposis colorectal cancer. In a prospective case-control study, we observed that significantly more colorectal cancer patients exhibited AEM than did age and sex-matched controls (19.5% *vs* 7.5%, $P<0.004$). However patients with AEMs do not have occult FAP, as we found no heterozygous adenomatous polyposis coli (*APC*) gene mutations despite extensive analysis of constitutional DNA. Genome-wide DNA replication errors (RERs) occur in a proportion of colorectal cancers, particularly right-sided lesions and in almost all tumours from hereditary non-polyposis colorectal cancer (HNPCC) patients. As AEMs have been reported in familial colon cancer cases, we investigated the relationship of AEMs to tumour RER phenotype. There was indeed an excess of AEMs in patients with right-sided tumours (30.2% of 53 patients *vs* 14.7% of 116 patients, $P<0.03$) and in those with RER tumours (3 out of 12 patients with RER tumours *vs* none out of 21 patients with non-RER tumours, $P<0.05$). Two patients with AEM were from HNPCC families compared with none of those without AEM ($P<0.05$). The association of AEMs with colorectal cancer is intriguing, and we speculate that it may be a manifestation of mutational mosaicism of the *APC* gene, perhaps associated with a constitutional defect in DNA mismatch pair.

**Keywords:** colorectal cancer; genetic instability; *APC* gene

An association has been reported between sporadic colorectal cancer and two clinical features that are normally considered to be extracolonic manifestations of the autosomal dominant syndrome familial adenomatous polyposis (FAP) (Sondergaard *et al.*, 1993; 1985*a*, *b*; Houlston *et al.*, 1992; Dunlop, 1983; Hunt *et al.*, 1994). These features, congenital hypertrophy of the retinal pigment epithelium (CHRPE) and mandibular osteomas, occur in 90% and 75% respectively of patients with classical FAP (Utsunomiya and Nakamura, 1975; Bulow *et al.*, 1984; Traboulsi *et al.*, 1987; Berk *et al.*, 1988; Chapman *et al.*, 1989; Giardiello *et al.*, 1991; Hodgson *et al.*, 1994; Wallis *et al.*, 1994). The finding of such extracolonic features in patients with non-FAP colorectal cancer is of considerable interest because of the central, and probably initiating, role of the gene for FAP (*APC*) in colorectal tumorigenesis. Constitutional and heterozygous mutation of the *APC* gene is responsible for the FAP syndrome (Groden *et al.*, 1991; Nishishio *et al.*, 1991; Nagase and Nakamura, 1993), while a high proportion of non-FAP colorectal adenomas and cancers have somatic *APC* mutations (Powell *et al.*, 1992; Ichii *et al.*, 1993; Nagasse and Nakamura, 1993). Inactivating *APC* mutations have also been noted in dysplastic aberrant crypt foci (Jen *et al.*, 1994; Smith *et al.*, 1984), which are believed to be the very earliest histological manifestation of colorectal neoplasia.

The available data suggest that the extracolonic manifestations of FAP, which we term attenuated extracholonic manifestations (AEMs), occur particularly in colorectal cancer patients with a family history of the disease (Sondergaard *et al.*, 1985*b*; Houlston *et al.*, 1992; Hunt *et al.*, 1994). Thus we wished to determine whether there was any link with hereditary non-polyposis colorectal cancer (HNPCC), which is responsible for 2-5% of all colorectal cancer cases. HNPCC gene carriers develop few colorectal adenomas but are at high risk of colorectal cancer, particularly of the right colon, as well as other cancers (Lynch *et al.*, 1993; Jass *et al.*, 1994). Causative germline mutations in the DNA mismatch repair (MMR) genes *hMSH2*, *hMLH1*, *hPMS1* and *hPMS2* (Liu *et al.*, 1996) have been identified in HNPCC families. Such mutations result in defective MMR (Parsons *et al.*, 1993; Umar *et al.*, 1994), manifest as instability of repetitive DNA in tumours (Ionov *et al.*, 1993; Aaltonen *et al.*, 1993; Thibodeau *et al.*, 1993; Peltomaki *et al.*, 1993; Lothe *et al.*, 1993; Aaltonen *et al.*, 1994; Bhattacharyya *et al.*, 1994; Liu *et al.*, 1995*a*) and termed replication error (RER) tumour phenotype. Somatic mutations in *GTBP*, another MMR gene, are responsible for the RER phenotype in some tumours (Drummond *et al.*, 1995; Palombo *et al.*, 1995; Papadopolous *et al.*, 1995). However, germline *GTBP* mutations have not been identified in any of the highly penetrant HNPCC families analysed so far (Papadopoulos *et al.*, 1995), although *GTBP* involvement in low-penetrance colorectal cancer susceptibility has not been excluded. The RER tumour phenotype is present in about 15% of sporadic colorectal cancers (Ionov *et al.*, 1993; Aaltonen *et al.*, 1993;Thidodeau *et al.*, 1993; Lothe *et al.*, 1993; Liu *et al.*, 1995*a*), 80-90% of which are in the proximal colon (Ionov *et al.*, 1993; Thibodeau *et al.*, 1993; Lothe *et al.*, 1993).

We have investigated the prevalance of AEMs in a prospective case-control study. In order to elucidate the underlying basis of this association, we evaluated AEM in relation to tumour location, family history and tumour RER phenotype to ascertain any link with MMR deficiency and HNPCC. As we have excluded the possibility of unrecognised cases of FAP by extensive *APC* gene mutation analysis, our

Correspondence: MG Dunlop
Received 4 March 1996; revised 1 July 1996; accepted 9 July 1996

observations are consistent with the hypothesis that AEMs are a manifestation of mutation mosaicism of the *APC* gene and may indicate genetically determined cases in a population of sporadic colorectal cancer patients.

### Methods

*Study population and pedigree ascertainment*

In order to obtain an unselected patient population group, we enrolled 169 patients prospectively after surgery for colorectal cancer in four Lothian hospitals. The four hospitals together serve the entire population of Lothian and admit essentially all patients in the region with colorectal cancer on an emergency and elective basis. Thus, the study population should be reasonably free of bias. Known FAP cases or patients with colonic multiple polyposis were excluded. Paired age and sex-matched healthy community controls with no personal cancer history were enrolled from patients registered with local Edinburgh medical practices that refer patients to three of the same four hospitals from which the cancer patients were recruited. Family histories were documented in a standard proforma and verified from statutory Scottish General Register Office records for first- and second-degree relatives with cause of death, where appropriate. Disease status for live relatives in Scottish kindreds and members of families resident outside Scotland was verified from clinical or pathology records. All personal data, family history and retinal and mandible findings together with data on tumour histology, staging and site were collated in a computer database. Local ethics committee approval was granted for all aspects of this study, including mandibular radiography, ophthalmoscopy with mydriasis and blood sampling.

*Retinal and mandible examinations*

Bilateral retinal examination was performed in 167 of the patients and 160 of the controls described above by indirect ophthalmoscopy (Model Omega 100, Heine, Germany), using a Nikon 20D lens after mydriasis by one of two opthalmologists. All efforts were made to ensure that the ophthalomologists were unaware of the subjects' health status and did not discuss recent operations or family history with the patients. Retinal lesions were documented on fundal charts and photographed. Thirty patients (with and without CHRPE) were assessed by both ophthalomologists, and this ensured inter-observer reproducibility. The diagnostic criteria we used for retinal pigmentation in non-polyposis colorectal cancer patients were the same as those taken as diagnostic of CHRPE in familial adenomatous polyposis, namely: $\geqslant 1$ pigmented lesions with depigmented halo; $\geqslant 1$ pigmented lesions over one optic disc diameter; $\geqslant 3$ small bilateral pigmented lesions without a halo or $>4$ small unilateral lesions (Traboulsi *et al.*, 1987; Chapman *et al.*, 1988; Berk *et al.*, 1988; Giardello *et al.*, 1991; Hodgson *et al.*, 1994; Wallis *et al.*, 1994). Because of the variety of CHRPE lesions, we devised a scoring system derived from the relative importance ascribed to each feature in previous FAP studies. Pigmented haloed lesions and lesions $>1$ disc diameter scored 5: small lesions scored 1 and the total score for bilateral lesions was doubted.

Posteroanterior, occlusal and pantomographic radiographic views of the mandible were obtained using Roentgen 501, Siemens Heliodent MD and Morita Panex EC machines respectively on 163 patients and 159 controls, with immediate reveiw and further oblique or intra-oral views for inconclusive cases. Radiographs were interpreted independently on two separate occassions by a radiologist and an oral surgeon blinded to the subjects' disease status. Osteomas were defined as discrete homogeneous radio-opaque areas $>2$ mm in diameter with no surrounding radiolucent zone, distinct from the teeth and apices (Bulow *et al.*, 1984; Sondergaard *et al.*, 1985*a*, *b*; 1993).

APC *gene screening*

We wished to determine whether any of our findings could be explained on the basis of the constitutional and heterozygous *APC* mutations associated with a weakly expressed form of FAP. Hence, we searched for truncating *APC* mutations in all coding sequences from exon 9 to the termination signal at the 3′ end of exon 15, which includes all regions of *APC* known to be associated with expression of the CHRPE phenotype in FAP (Olschwang *et al.*, 1993). Peripheral blood from all 33 patients exhibiting AEM was screened by an *in vitro* synthesised protein assay (IVSP) (Powell *et al.*, 1993; Prosser *et al.*, 1994) for translation-terminating *APC* gene mutations and by heteroduplex analysis (Prosser *et al.*, 1994; Keen *et al.*, 1991) for each of exons 9–14. Positive control samples from FAP patients and manufacturer's control reactions were always run in parallel. The *APC* gene was amplified by polymerase chain reaction (PCR) in overlapping fragments from DNA and RNA purified from peripheral blood with forward PCR primers, including signals for transcription and translation. RNA was available for five patients in whom exons 1–14 were analysed by IVSP of reverse-transcribed complementary DNA as described (Prosser *et al.*, 1994). Resultant PCR products were used in a coupled transcription–translation reaction (Promega, UK) generating radiolabelled synthetic polypeptide sequences, which were analysed by SDS–PAGE followed by autoradiography. Approximately 500 bp of the most 5′ part of *APC* exon 15 was analysed by heteroduplex analysis in overlapping fragments using the PCR primers as described (Groden *et al.*, 1991) to exclude any mutations occurring in the most 5′ region of exon 15, which would result in such a short peptide in the IVSP assay that a mutation could be masked. Exons 9–14 were analysed separately in all 33 patients by heteroduplex analysis of PCR-amplified leucocyte DNA with electrophoresis on MDE™ gels (JT Baker, USA) and ethidium bromide staining (Keen *et al.*, 1991). Any heteroduplex variants between normal and tumour DNA were reamplified and sequenced.

*Tumour DNA instability analysis*

As microsatellite DNA instability (RER phenotype) has been observed in colorectal cancers and is the result of inherited or of somatic mutation in one of the DNA mismatch repair genes (Leach *et al.*, 1993; Parsons *et al.*, 1993; Liu *et al.*, 1995*a*; Papadopoulos *et al.*, 1995), we wished to correlate our findings of the AEM phenotype with such tumour DNA instability. We were able to obtain fresh surgical resection specimens form 33 of the patients recruited to the study at one of the participating hospitals (12 with and 21 without AEMs). DNA was purified from tumour and normal mucosa and RER status assessed by comparison of PCR-amplified paired tumour/control DNAs at six PCR-amplified $(CA)_n$ repeat marker loci: D2S123, D2S119, D3S1293, D8S282, D13S160 (Gyapay *et al.*, 1994) and at a polyadenine tract; BAT40 (Liu *et al.*, 1995*b*). Formamide-denatured PCR products were electrophoresed on 6% denaturing acrylamide gels in 0.5×TBE buffer and silver-stained (Bassam *et al.*, 1991). All aberrant banding patterns indicative of RER phenotype were confirmed by repeat analyses. Tumours were considered to exhibit the RER phenotype when band alterations were present at two or more loci when compared with matched control DNA as described previously (Liu *et al.*, 1995*a*, *b*).

### Results

*Study group characteristics and family history*

Cancer patients and control subjects were well age and sex matched, although some paired control subjects did not attend. There were 71 females and 98 male patients (mean age 63.1 years, range 20–92 years) and 69 female and 91 male controls (mean age 62.4 years, range 32–86 years). One hunded and fifty-one patients (89%) and 141 controls (87%) were of Scottish descent. More patients than controls had first-degree relatives with colorectal cancer (40/169, 24% *vs* 16/162, 10%; $P<0.003$). The index case was excluded to avoid bias in the ascertainment of HNPCC families, as, by definition, controls cannot contribute a colorectal cancer case. Two of the patient kindreds (1.2%) fulfilled HNPCC criteria (Vasen *et al.*, 1991) but none of the control families did so. Sixteen patient (9.5%) and no control families ($P<0.0002$) fulfilled more relaxed criteria for genetically determined cases ($\geqslant 2$ colorectal and $\geqslant 1$ other HNPCC cancers in a first-degree kinship) (Mecklin, 1987; Kee and Collins, 1991).

*Prevalence of AEM in colorectal cancer patients*

The prevalence of retinal and/or mandibular extracolonic manifestations of FAP was significantly higher in patients than in controls (33/169, 19.5% *vs* 12/160, 7.5%; $P<0.004$), and the major contribution to this excess was retinal pigmentation. Summary data of the proportion of each patient group with AEMs are shown in Table I. Although we assessed a reasonably large study population at the outset and the differences that we observed do reach statistical significance, it should be noted that the numbers in some of the subcategories are small. Nonetheless, there does appear to be an association of AEMs with colorectal cancer and, within the cancer group, with proximal tumour location, with an RER phenotype and with a family history of HNPCC.

A representative case of multiple small patches of retinal pigmentation is shown in Figure 1. Although the features were similar to that seen in FAP, they were of lower magnitude, being generally smaller, and there were none of the typical large 'bear-track' lesions seen in FAP (Berk *et al.*, 1988; Hodgson *et al.*, 1994). However, there was a substantial excess of patients with retinal pigmentation, as 22/167 patients (13%) *vs* 8/159 control subjects (5%) had lesions which fulfilled published diagnostic criteria for FAP patients (Berk *et al.*, 1988; Chapman *et al.*, 1988; Wallis *et al.*, 1944; Hodgson *et al.*, 1994) ($P<0.02$). Retinal pigmentation expressed as CHRPE score for both patients and controls is shown in Figure 2. In a sign test comparison of matched patient/control pairs, patient CHRPE scores were significantly greater than those of controls (Figure 2). For this analysis, there were 160 pairs as not all control subjects attended, and so the corresponding paired patients were excluded. Patient scores exceeded controls in 45 and were less than controls in 22 comparisons ($P<0.01$). Both patients from HNPCC families had CHRPE, and 32% of patients with CHRPE had a first-degree relative affected by bowel cancer.

One of the control subjects with striking retinal pigmentation had a father and aunt with colorectal cancer. In the control group as a whole, in which we were able to confirm a negative family history, CHRPE prevalence was substantially less than in the colorectal cancer patients (4.9% *vs* 19.5%, $P<0.02$).

Osteoma prevalence was almost 3-fold greater in patients than in control subjects, although osteoma prevalence alone did not reach statistical significance owing to the small numbers. Thirteen of 163 patients (8.0%) and 5 of 159 (3%) controls undergoing mandibular radiography had $\geqslant 1$ osteoma. An orthopantomogram from a patient with colorectal cancer is shown in Figure 3.

**Table I** Prevalence of extracolonic manifestations of FAP (AEMs) in patients with sporadic colorectal cancer and in healthy control subjects. Relationship of AEMs to patient family history of HNPCC, tumour location and tumour RER phenotype

| | n | *Individuals with AEM* |
|---|---|---|
| Healthy control subjects | 160 | 12 (7.5%) |
| Colorectal cancer patients | 169 | 33 (19.5%) |
| Right-sided tumour | 53 | 16 (30.2%)* |
| Distal tumour | 116 | 17 (14.7%)* |
| RER tumour phenotype | 12 | 3 (25%) |
| Non-RER tumour | 21 | 0 |
| FH[a] of HNPCC | 2 | 2 (6%) |
| No FH[a] of HNPCC | 31 | 0 |

[a]FH, family history. *$P<0.05$.

*APC gene mutation analysis*

There were no constitutional truncating *APC* mutations identified in peripheral blood DNA for RNA in 33 patients with AEM (data not shown). The combination of IVSP assays of exon 15 and heteroduplex analysis of exons 9–14 for all patients, along with IVSP analysis of cDNA for exons 1–14 in five patients, excludes the presence of heterozygous truncating *APC* mutations in those patients with extracolonic features of FAP. Hence, unrecognised cases of FAP cannot explain these observations.

*Tumour clinicopathological characteristics*

There were 172 carcinomas in 169 patients, three patients (1.8%) having synchronous tumours. There were 14 Dukes'

**Figure 1** Fundal photograph of one sector of the retina in a patient with colorectal cancer and retinal pigmentation (arrowed). There are several small pigmented areas in this and other retinal quadrants and also a small number in the contralateral eye.

**Figure 2** Plot of CHPRE scores from cases and controls demonstrating that a significantly greater proportion of patients had CHRPE scores ⩾5, corresponding to previous CHRPE diagnostic criteria (22/167, 13% *vs* 8/159, 5%; $P<0.02$).

**Figure 3** Orthopantomogram from a patient with colorectal cancer with several radiodense regions in the body of the mandible (arrowed).

stage A (8.1%), 85 stage B (49.4%), 73 stage C (42.5%). There was an association between the presence of AEMs and right-sided tumours (Table II). Right-sided tumours have also been shown to be associated with a RER phenotype (Ionov *et al*., 1993; Thibodeau *et al*., 1993; Lothe *et al*., 1993; Kim *et al*., 1994). Sixteen of 53 patients (30.2%) with right (i.e. caecal, ascending and hepatic flexure) colonic tumours had AEMs compared with 17 of 116 patients (14.7%) with more distal lesions ($P<0.032$). Interestingly, two of the patients with synchronous tumours (66%) had AEMs while the third had two small CHRPE lesions.

### *Tumour DNA instability and AEM*

We analysed DNA from a total of 33 tumours for the presence of genetic instability, 12 from patients with AEMs and 21 from patients without AEMs. In all, three tumours exhibited an RER phenotype, and all of these tumours were from patients with AEMs. Thus, three tumours from the 12 patients (25%) and none from the 21 patients without AEMs were RER tumours ($P<0.05$). Representative silver-stained gels demonstrating the RER phenotype at the marker locus D13S160 in three of the four tumours are shown in Figure 4. Although we did not consider band shifts at only one locus to be diagnostic of RER phenotype, four of the seven patients (57%) with tumours exhibiting RER at ⩾1 locus had AEMs, and the tumour was proximal in all of these cases.

**Table II** Location of 172 colorectal cancers in 169 patients and AEM status. There were three patients with a total of two synchronous tumours each, and these are discussed in the text

| *Anatomical site* | *Number (%) of tumours (n = 172)* | *Number (%) of patients with AEMs* |
|---|---|---|
| Caecum | 31 (18%) | 6 (19.4%) |
| Ascending colon | 15 (8.2%) | 7 (46.7%) |
| Hepatic flexure | 7 (4.1%) | 3 (42.9%) |
| Transverse colon | 10 (5.8%) | 1 (10.0%) |
| Splenic flexure | 6 (3.5%) | 2 (33.3%) |
| Descending colon | 8 (4.7%) | 1 (12.5%) |
| Sigmoid | 34 (20%) | 6 (17.6%) |
| Rectum | 61 (35.7%) | 10 (16.4%) |

**Figure 4** Representative silver-stained amplification products after acrylamide gel electrophoresis for the marker D13S160 from tumour and normal mucosa DNA from four patients. Novel, tumour specific bands are present in tumours A, B and D indicating tumour DNA instability. In tumour A there has been a contraction from normal in the repeat DNA at the D13S160 locus, whereas in tumour B there has been an expansion. Tumour and normal are identical at the D13S160 locus in patient C.

### Discussion

The results of this case–control study establish the association between colorectal cancer and extracolonic features normally associated with the autosomal dominant syndrome of FAP. Our observations support and extend previous reports that have noted extracolonic manifestations of FAP in patients with colorectal cancer (Sondergaard *et al*., 1985*a*; 1993; Houlston *et al*., 1992; Hunt *et al*., 1994). An association between sporadic colorectal cancer and mandibular osteomas has been reported in Scandinavia (Sondergaard *et al*., 1985*a*; *b*; 1993), and an excess of CHRPE in selected patients with familial non-polyposis colorectal cancer has been reported in the UK (Houlston *et al*., 1992; Hunt *et al*., 1994). Our findings accord well with UK study of Hunt *et al*. (1994), in which a lower patient prevalence of mandibular osteomas than that reported in Scandinavia (Sondergaard *et al*., 1985*a*; 1993) was observed. These population differences may indicate the involvement of environmental factors. In a recent study by the same Scandinavian authors, there was no evidence of an excess of CHRPE in a small cohort of 34 colorectal cancer patients (Hartvigsen *et al*., 1995). However, patients with a family history of colorectal cancer were specifically excluded from that study and so comparison with our findings is not possible. Nonetheless, taken together, these observations have substantial clinical relevance and suggest that currently healthy individuals with retinal pigmentation or craniofacial osteomas typical of AEMs may be at increased risk of colorectal cancer. Further studies are required to determine if the controls in this study who had evidence of AEMs have a higher than expected incidence of colorectal neoplasia.

The first of two lines of investigation that we pursued, to elucidate the molecular basis of the observed excess of AEM

in colorectal cancer patients, was to exclude the presence of constitutional *APC* gene mutations, as some cases might be due to unrecognised FAP. We excluded such heterozygous *APC* mutations as far as is reasonably practical in all cancer patients with AEMs. *APC* mutations associated with expression of the CHRPE phenotype in FAP patients are restricted to a segment downstream of exon 8 (Olschwang *et al.*, 1993). We employed sensitive mutation detection techniques to carry out a detailed analysis of this entire region in blood leucocyte DNA or RNA from all patients exhibiting the AEM phenotype (Powell *et al.*, 1993; Prosser *et al.*, 1994). Constitutional *APC* gene defects upstream of intron 4 can induce an attenuated FAP phenotype with very few polyps and the late onset of cancer (Spirio *et al.*, 1993). Although mutations in exons 1–4 could be responsible for some cases of apparently sporadic colorectal cancer, CHRPE is never present in FAP cases with such mutations (Olschwang *et al.*, 1993) and so cannot explain our findings. Another explanation that seems improbable concerns the possibility that constitutional *APC* mutations resulting only in amino acid alterations could be responsible for both AEM and colorectal cancer susceptibility. However, mutations resulting in protein truncation or in substantial reduction in intracellular APC protein concentration is an absolute requirement for the development of FAP (Nagase and Nakamura, 1993; Powell *et al.*, 1993). In addition, truncating mutations are found almost universally in sporadic colorectal carcinomas (Nagase and Nakamura, 1993; Powell *et al.*, 1992), adenomas (Powell *et al.*, 1992; Ichii *et al.*, 1993) and even in some aberrant crypt foci (Jen *et al.*, 1994; Smith *et al.*, 1994). This argues strongly that mis-sense APC mutations are not responsible for our findings of an association of colorectal neoplasia and AEMs.

Next, we determined the proportion of patients with AEMs that exhibited genetic instability, as mutations in mono- and dinucleotide anonymous repeat sequence mutations have been observed in normal tissues in a subset of HNPCC patients with a tumour RER phenotype (Parsons *et al.*, 1995). Although the numbers of cases in some of the subcategories were small, we did observe a statistically significant association between AEMs and a family history of HNPCC, a proximal tumour preponderance and tumour DNA instability, all of which are very suggestive of the involvement of defective DNA mismatch repair. The fact that not all patients with AEMs had a tumour with the RER phenotype may be due to failure of our RER assay to identify subtle DNA repair defects resulting from particular mutations in the known *MMR* genes *hMSH2*, *hMLH1*, *hPMS1* and *hPMS2*. It is also possible that defects within other genes known to participate in DNA mismatch repair may induce a weak, constitutional mutator phenotype. One such obvious candidate is *GTBP*, for which no germline mutations have been found in large classical HNPCC families (Papadopoulos *et al.*, 1995), but low-penetrance mutations in *GTBP* may be responsible for a proportion of apparently sporadic colorectal cancer. Several other genes homologous with the known *MMR* genes are also currently under investigation by a number of research groups. Such genes may result in a low level of genetic instability, which could explain the lack of tumour RER phenotype in all patients with AEMs.

There is already evidence to support the notion that defective DNA mismatch repair can result in a constitutional mutator phenotype and that such a phenotype results in predisposition to cancer. Genetic instability has been observed in normal tissue and sperm from transgenic mice with homozygous inactivation of *MLH1* and *MSH2* (de Wind *et al.*, 1995; Baker *et al.*, 1995). The constitutional mutator phenotype that has been observed in HNPCC patients with only a heterozygous *MMR* gene mutation (Parsons *et al.*, 1995) suggests that, in some cases, a dominant-negative effect may be involved. These previous studies set the precedent that *MMR* deficiency could induce somatic mutational mosaicism at simple repeat arrays. We suggest that such somatic mosaicism could also involve mutation at the *APC* gene. Such mutational mosaicism would be expected to be associated with an elevated colorectal cancer risk in addition to the occurence of other features of FAP, albeit less frequently and to a lesser degree than in true FAP cases. Thus, we propose that the association of AEM and DNA instability reported here is best explained on the basis of *APC* mutations occurring in a small proportion of all *normal* somatic cells, including cells contributing to colorectal mucosa, retinal pigment epithelium and osseous tissue. This explanation invokes the notion of *APC* mutational mosaicism induced by DNA instability.

Further support for a link between extracolonic features of FAP and susceptibility to colorectal cancer mediated by genetic instability comes from three different sources. One descriptive report documents a family in which several family members were affected by colorectal cancer (Maher *et al.*, 1992). In this family, there were also a number of relatives with fibrous tumours typical of desmoid disease, a feature well described in classical FAP and affecting 5–7% of polyposis patients. Further supporting evidence concerns the association of brain tumours with colonic polyposis in Turcot's syndrome. The genetic basis of Turcot's syndrome in most cases is germline *APC* mutation, identical to the mutations seen in classical FAP (Hamilton *et al.*, 1995). However, in a minority of cases, germline mutation of a mismatch repair gene, not *APC*, is responsible, and this is accompanied by a RER tumour phenotype. Finally, it is now clear that the RER phenotype does have an important influence from the very earliest stages of neoplastic transformation in the colorectum. Our own recent findings indicate that genetic instability has a marked influence on the occurrence of *APC* mutations during neoplastic transformation in the colorectal epithelium (Huang *et al.*, 1996). In that study, we found that simple repeats within the *APC* gene, especially poly-A-tracts, appear to be particular targets for replication slippage mutations in tissues that are defective in *MMR*. Thus normal tissues, including the germline, in patients with relative *MMR* deficiency might also acquire mutations in the *APC* gene. Somatic mutational mosaicism has been observed in rare cases in other heritable cancer syndromes, including Li–Fraumeni syndrome (Kovar *et al.*, 1992), retinoblastoma (Greger *et al.*, 1990) and Wilms' tumour (Chao *et al.*, 1993).

Mosaicism may explain why the retinal and mandibular changes that we observed in this study group were more subtle that those seen in FAP patients. Despite every cell carrying a mutant *APC* alllele in FAP patients, only a small proportion of cells contribute to retinal and osseous lesions. Indeed, even in the colorectum, there are some normal cells; and some FAP patients have relatively few colonic adenomas. Our proposed model implies that only a small proportion of retinal and mandibular cells carry a mutant *APC* allele, and so the number and extent of the retinal, osseous and indeed colorectal lesions would be expected to be proportionately less. Such mosaicism could represent a mechanism by which mutations in expressed genes involved in cancer development could accumulate in stem cells in adult, or even in intrauterine, life without a requirement for induction of clonal expansion. Thus, the degree of mandibular and retinal changes would be indicative of the level of mosaicism and perhaps even cancer risk. It is intriguing that two of the three patients with synchronous tumours had AEM while the third also had two small CHRPE lesions. Definitive proof of somatic *APC* mosaicism in patients with AEM will require further extensive detailed molecular analysis.

Although we favour *APC* mutational mosaicism as the best interpretation of our findings, other possible hypotheses should be considered. The features noted here could represent phenocopies of FAP manifestations or be due to constitutional down-regulation of *APC* by a modifier gene such as *Mom 1* (Dietrich *et al.*, 1993). However, these interpretations do not addresss our observations of a familial tendency, the greater proportion of patients with right-sided tumours exhibiting AEMs and the association of AEM with tumour DNA instability. The strong family history of colorectal cancer in a control subject with marked retinal pigmentation is also suggestive of a real association between AEM and familial cancer susceptibility.

In conclusion, we have demonstrated an excess of extracolonic features of FAP in an unselected patient group with colorectal cancer but without the colonic polyposis characteristic of FAP or a constitutional mutation in one allele of the *APC* gene. Although the relationship is not exclusive, the AEM phenotype does appear to be linked with proximal tumour location, the RER tumour phenotype and with a family history of HNPCC, all of which are known to be associated with defective DNA mismatch repair. Further studies are required to investigate these novel observations and to elucidate this potential role for *MMR* deficiency in cancer predisposition. In addition, follow-up of healthy individuals found to have AEM in ths study may determine whether there is indeed an associated increase in colorectal cancer risk and whether the presence of AEM merits colonoscopic surveillance.

**Acknowledgements**

We thank N Brown for clinical organisation, R De Mey and A Fordyce for genealogy, A Carrothers for statistical advice, M Phillips for oral radiography and the general practitioners of Muirhouse and Blackhall for access to matched control subjects. In particular, we thank all patient and control subjects who participated in this study. This work was supported by grants from the Cancer Research Campaign (SP2326/0101, SP1370/0501), Scottish Hospitals Endowment Research Trust (SHERT 1042), Edinburgh University Cancer Research Fund, Sir Stanley and Lady Davidson Fund and the Melville Trust.

### References

AALTONEN LA, PELTOMAKI P, LEACH FS, SISTONEN P, PYLKKANEN L, MECKLIN J-P, JARVINEN H, POWELL SM, JEN J, HAMILTON SR, PETERSEN GM, KINZLER KW, VOGELSTEIN B AND DE LA CHAPELLE A. (1993). Clues to the pathogenesis of familial colon cancer. *Science*, **260**, 812–816.

AALTONEN LA, PELTOMAKI P, MECKLIN JP, JARVINEN H, JASS JR, GREEN JS, LYNCH HT, WATSON P, TALLQVIST G, JUHOLA M, SISTONEN P, HAMILTON SR, KINZLER KW, VOGELSTEIN B AND DE LA CHAPELLE A. (1994). Replication errors in benign and malignant tumors from herediatry non-polyposis colorectal cancer patients. *Cancer Res.*, **54**, 1645–1648.

BAKER SM, BRONNER CE, ZHANG L, PLUG AW, ROBATZEK M, WARREN G, ELLIOT EA, YU J, ASHLEY T, ARNHEIM N, FLAVELL RA AND LISKAY RM. (1995). Male mice defective in the DNA mismatch repair gene PMS2 exhibit abnormal chromosome synapsis in meiosios. *Cell*, **82**, 309–319.

BASSAM BJ, CAETANO-ANOLLES G AND GRESSHOFF PM. (1991). Fast and sensitive silver staining of DNA in polyacrylamide gels. *Anal. Biochem.*, **196**, 80–83.

BERK T, COHEN Z, MCLEOD RS AND PARKER JA. (1988). Congenital hypertrophy of the retinal pigment epithelium as a marker for familial adenomatous polyposis. *Dis. Colon Rectum*, **31**, 253–257.

BHATTACHARYYA NP, SKANDALIS A, GRODEN J AND MEUTH M. (1994). Mutator phenotypes in human colorectal carcinoma cell lines. *Proc. Natl Acad. Sci. USA*, **91**, 6319–6323.

BULOW S, SONDERGAARD JO, WITT IN, LARSEN E AND TETENS G. (1984). Mandibular osteomas in familial polyposis *coli*. *Dis. Colon Rectum*, **27**, 105–108.

CHAO LY, HUFF V, TOMLINSON G, RICCARDI VM, STRONG LC AND SAUNDERS GF. (1993). Genetic mosaicism in normal tissues of Wilms' tumour patients. *Nature Genet.*, **3**, 127–131.

CHAPMAN PD, CHURCH W, BURN J AND GUNN A. (1989). The detection of congenital hypertrophy of the retinal pigment epithelium (CHRPE) by indirect ophthalmoscopy; a reliable clinical feature of familial adenomatous polyposis. *Br. Med. J.*, **298**, 353–354.

DIETRICH WF, LANDER ES, SMITH JS, MOSER AR, GOULD KA, LUONGO C, BORENSTEIN N AND DOVE W. (1993). Genetic identification of Mom-1, a major modifier locus affecting Min-induced intestinal neoplasia in the mouse. *Cell*, **75**, 631–639.

DRUMMOND J, LI G-M, LONGLEY M AND PAUL MODRICH. (1995). Isolation of an hMSH2-p160 heterodimer that restores DNA mismatch repair to tumor cells. *Science*, **268**, 1909–1912.

DUNLOP MG. (1993). Inheritance of colorectal cancer susceptibility. *Br. J. Surg.*, **77**, 245.

GIARDIELLO FM, OFFERHAUS GJ, TRABOULSI EI, GRAYBEAL JC, MAUMENEE IH, KRUSH AJ, LEVIN LS, BOOKER SV AND HAMILTON SR. (1991). Value of phenotypic markers in identifying inheritence of familial adenomatous polyposis. *Gut*, 1170–1174.

GREGER V, PASSARGE E AND HORSTHEMKE B. (1990). Somatic mosaicism in a patient with bilateral retinoblastoma. *Am. J. Hum. Genet.*, **46**, 1187–1193.

GRODEN J, THLIVERIS A, SAMOWITZ W, CARLSON M, GELBERT L, ALBERTSON H, JOSLYN G, STEVENS J, SPIRIO L, ROBERTSON M, SARGEANT L, KRAPCHO K, WOLFF E, BURT R, HUGHES JP, WARRINGTON J, MCPHERSON J, WASMUTH J, LE PASLIER D, ABDERRAHIM H, COHEN D, LEPPERT M AND WHITE R. (1991). Identification and characterization of the familial adenomatous polyposis coli gene. *Cell*, **66**, 589–600.

GYAPAY G, MORISSETTE J, VIGNAL A, DIB C, FIZAMES C, MILLASSEAU P, MARC S, BERNARDI G, LATHROP M AND WEISSENBACH J. (1994). The 1993–94 Genethon Human Linkage Map. *Nature Genet.*, **7**, 246–339.

HAMILTON SR, LIU B, PARSONS RE, PAPADOPOULOS N, JEN J, POWELL SM, KRUSH AJ, BERK T, COHEN Z, TETU B, BURGER PC, WOOD PA, TAQI F, BOOKER S, PETERSEN GM, OFFERHAUS GJA, TERSMETTE AC, GIARDIELLO FM, VOGELSTEIN B AND KINZLER KW. (1995). The molecular basis of Turcot's syndrome. *N. Engl. J. Med.*, **332**, 839–847.

HARTVIGSEN A, MYRHOJ T, BULOW S, BORME KK AND SONDERGAARD JO. (1995). Ophthalmoscopy for congenital hypertrophy of the retinal pigment epithelium (CHRPE) in patients with sporadic colorectal carcinoma. *Int. J. Colorect. Dis.*, **10**, 138–139.

HODGSON SV, BISHOP DT AND JAY B. (1994). Genetic heterogeneity of congenital hypertrophy of the retinal pigment epithelium (CHRPE) in families with familial adenomatous polyposis. *J. Med. Genet.*, **31**, 55–58.

HOULSTON RS, FALLON T, HARCOPOS C, WILLIAMS CB, DAVEY C AND SLACK J. (1992). Congenital hypertrophy of retinal pigment epithelium in patients with colonic polyps associated with cancer family syndrome. *Clin. Genet.*, **42**, 16–18.

HUANG J, PAPADAPOULOS N, MCKINLEY AJ, FARRINGTON SM, CURTIS LJ, WYLLIE AH, ZHENG S, WILLSON JKV, MARKOWITZ SD, MORIN P, KINZLER KW, VOGELSTEIN B AND DUNLOP MG. (1996). APC mutations in colorectal tumours with mismatch repair deficiency. *Proc. Natl. Acad. Sci.*, **93**, 9049 – 9054..

HUNT LM, ROBINSON MH, HUGKULSTONE CE, CLARKE B, VERNON SA, GREGSON RH, HARDCASTLE JD AND ARMITAGE NC. (1994). Congenital hypertrophy of the retinal pigment epithelium and mandibular osteomata as markers in familial colorectal cancer. *Br. J. Cancer*, **70**, 173–176.

ICHII S, TAKEDA S, HORII A, NAKATSURU S, MIYOSHI Y, EMI M, FUJIWARA Y, KOYAMA K, FURUYAMA J, UTSONOMIYA J AND NAKAMURA Y. (1993). Detailed analysis of genetic alterations in colorectal tumours from patients with and without familial adenomatous polyposis (FAP). *Onocgene*, **8**, 2399–2405.

IONOV Y, PEINADO MA, MALKHOSYAN S, SHIBATA D AND PERUCHO M. (1993). Ubiquitous somatic mutations in simple repeated sequences reveal a new mechanisms for colonic carcinogenesis. *Nature*, **363**, 558–561.

JASS JR, STEWART SM, STEWART J AND LANE MR. (1994). Hereditary non-polyposis colorectal cancer-morphologies, genes and mutations. *Mutat. Res.*, **310**, 125–133.

JEN J, POWELL SM, PAPADOPOULOS N, SMITH KJ, HAMILTON SR, VOGELSTEIN B AND KINZLER KW. (1994). Molecular determinants of dysplasia in colorectal lesions. *Cancer Res.*, **54**, 5523–5526.

KEE F AND COLLINS BJ. (1991). How prevalent is cancer family syndrome? *Gut*, **32**, 509 – 512.

KEEN J, LESTER D, INGLEHEARN C, CURTIS A AND BHATTACHARYA S. (1991). Rapid detection of single-base mismatches as heteroduplexes on Hydrolink gels. *Trends Genet.*, **7**, 5.

KIM H, JEN J, VOGELSTEIN B AND HAMILTON SR. (1994). Clinical and pathological characteristics of sporadic colorectal carcinomas with DNA replication errors in microsatellite sequences. *Am. J. Pathol.*, **145**, 148–156.

KOVAR H, AUINGER A, JUG G, MULLER T AND PILLWEIN K. (1992). p53 mosaicism with an exon 8 germline mutation in the founder of a cancer-prone pedigree. *Oncogene*, **7**, 2169–2173.

LEACH FS, NICOLAIDES NC, PAPADOPOLOUS N, LIU B, JEN J, PARSONS R, PELTOMAKI P, SISTONEN P, AALTONEN LA, NYSTROM-LAHTI M, GUAN X-Y, ZHANG J, MELTZER PS, YU J-W, KAO F-T, CHEN DJ, CEROSALETTI KM, FOURNIER REK, TODD S, LEWIS T, LEACH RJ, NAYLOR SL, WEISSENBACH J, MECKLIN J-P, JARVINEN H, PETERSEN GM, HAMILTON SR, GREEN J, JASS J, WATSON P, LYNCH HT, TRENT JM, DE LA CHAPELLE A, KINZLER KW AND VOGELSTEIN B. (1993). Mutations of a MutS homolog in hereditary non-polyposis colorectal cancer. *Cell*, **75**, 1215–1225.

LIU B, FARRINGTON SF, PETERSEN GM, HAMILTON SR, PARSONS R, PAPADOPOULOS N, FUJIWARA T, JEN J, KINZLER KW, WYLLIE AH, VOGELSTEIN B AND DUNLOP MG. (1995*a*). Genetic instability in the majority of young patients with colorectal cancer. *Nature Med.*, **1**, 348–352.

LIU B, PARSONS R, PAPADOPOULOS N, NICOLAIDES NC, LYNCH HT, WATSON P, JASS JR, DUNLOP MG, WYLLIE AH, JESSUP JM, PELTOMAKI P, DE LA CHAPELLE A, HAMILTON SR, VOGELSTEIN B AND KINZLER KW. (1996). Analysis of mismatch repair genes in hereditary non-polyposis colorectal cancer patients. *Nature Med.*, **2**, 169–174.

LIU B, NICOLAIDES NC, MARKOWITZ S, WILLSON JKV, PARSONS RE, JEN J, PAPADOPOULOS N, PELTOMAKI P, DE LA CHAPELLE A, HAMILTON SR, KINLZER KW AND VOGELSTEIN B. (1995*b*) Mismatch repair gene defects in sporadic colorectal cancers with microsatellite instability. *Nature Genet.*, **9**, 48–55.

LOTHE RA, PELTOMAKI P, MELING GI, AALTONEN LA, NYSTROM-LAHTI M, PYLKKANEN L, HEIMDAL K, ANDERSEN TI, MOLLER P, ROGNUM TO, FOSSA SD, HALDORSEN T, LANGMARK F, BRUGGER A, DE LA CHAPELLE A AND BORRESEN A-L. (1993). Genomic instability in colorectal cancer: relationship to clinicopathological variables and family history. *Cancer Res.*, **53**, 5849–5852.

LYNCH HT, SMYRK TC, WATSON P, LANSPA SJ, LYNCH JF, LYNCH PM, CAVELIERI RJ AND BOLAND CR. (1993). Genetics, natural history, tumor spectrum, and pathology of hereditary nonpolyposis colorectal cancer: an updated review. *Gastroenterology*, **104**, 1535–1549.

MAHER ER, MORSON B, BEACH R AND HODGSON SV. (1992). Phenotypic variation in hereditary nonpolyposis colon cancer syndrome. Association with infiltrative fibromatosis (desmoid tumour). *Cancer*, **69**, 2049–2051.

MECKLIN J-P. (1987). Frequency of hereditary colorectal carcinoma. *Gastroenterology*, **93**, 1021–1025.

NAGASE H AND NAKAMURA Y. (1993) Mutations of the APC (adnematous polyposis coli) gene. *Hum. Mutat.*, **2**, 425–434.

NISHISHO I, NAKAMURA Y, MIYOSHI Y, MIKI Y, ANDO A, HORRI A, KOYAMA K, UTSUNOMIYA J, BABA S, HEDGE P, MARKHAM A, KRUSH AJ, PETERSEN GM, HAMILTON SR, NIBERT MC, LEVY DB, BRYAN TM, PREISINGER AC, SMITH KJ, SU L-K, KINZLER KW AND VOGELSTEIN B. (1991). Mutations of chromosome 5q21 genes in FAP and colorectal cancer patients. *Science*, **253**, 665–669.

OLSCHWANG S, TIRET A, LAURENT-PUIG P, MULERIS M, PARC R AND THOMAS G. (1993). Restriction of ocular fundus lesions to a specific subgroup of APC mutations in adenomatous polyposis coli patients. *Cell*, **75**, 959–968.

PALOMBO F, GALLINARI P, IACCARINO I, LETTIERI T, HUGHES M, D'ARRIGO A, TRUONG O, HSUAN JJ AND JIRICNY J. (1995). GTBP, a 160-kilodalton protein essential for mismatch-binding activity in human cells. *Science*, **268**, 1912–1914.

PAPADOPOLOUS N, NICOLAIDES NC, LIU B, PARSONS R, LENGAUER C, PALOMBO F, D'ARRIGO A, MARKOWITZ S, WILLSON JK, KINZLER KW, JIRICNY J AND VOGELSTEIN B. (1995). Mutations of GTBP in genetically unstable cells. *Science*, **268**, 1915–1917.

PARSONS R, LI G-M, LONGLEY MJ, FANG W-H, PAPADOPOLOUS N, JEN J, DE LA CHAPELLE A, KINZLER KW, VOGELSTEIN B AND MODRICH P. (1993). Hypermutability and mismatch repair dificiency in $RER^{+}$ tumor cells. *Cell*, **75**, 1227–1236.

PARSONS R, LI G-M, LONGLEY M, MODRICH P, LIU B, BERT T, HAMILTON SR, KINZLER KW AND VOGELSTEIN B. (1995). Mismatch repair deficiency in phenotypically normal human cells. *Science*, **268**, 738–740.

PELTOMAKI P, LOTHE RA, AALTONEN LA, PYLKKANEN L, NYSTROM-LAHTI M, SERUCA R, DAVID L, HOLM R, RYBERG D, HAUGEN A, BRUGGER A, BORRESEN A-L AND DE LA CHAPELLE. (1993). Microsatellite instability is associated with tumors that characterize the hereditary non-polyposis colorectal carcinoma syndrome. *Cancer Res.*, **53**, 5853–5855.

POWELL SM, ZILZ N, BEAZER-BARCLAY Y, BRYAN TM, HAMILTON SR, THIBODEAU SN, VOGELSTEIN B AND KINZLER KW. (1992). APC mutations occur early during colorectal tumorigenesis. *Nature*, **359**, 235–237.

POWELL SM, PETERSEN GM, KRUSH AJ, BOOKER S, JEN J, GIARDIELLO FM, HAMILTON SR, VOGELSTEIN B AND KINZLER KW. (1993). Molecular diagnosis of familial adenomatous polyposis. *N. Engl. J. Med.*, **329**, 1982–1987.

PROSSER J, CONDIE A, WRIGHT M, HORN JM, FANTES JA, WYLLIE AH AND DUNLOP MG. (1994). APC mutation analysis by chemical cleavage of mismatch and a protein truncation assay in familial adenomatous polyposis. *Br. J. Cancer*, **70**, 841–846.

SMITH AJ, STERN HS, PENNER M, HAY M, MITRI A, BAPAT BV AND GALLINGER S. (1994). Somatic APC and K-ras codon 12 mutations in aberrant crypt foci from human colons. *Cancer Res.*, **54**, 5527–5530.

SONDERGAARD JO, SVEDSEN LB, WITT IN, BULOW S, LAURITSEN KB AND TETENS G. (1985*a*). Mandibular osteomas in colorectal cancer. *Scand. J. Gastoenterol.*. **20**, 759–761.

SONDERGAARD JO, SVEDSEN LB, WITT IN, BULOW S, LAURITSEN KB AND TETENS GB. (1985*b*). Mandibular osteomas in the cancer family syndrome. *Br. J. Cancer*, **52**, 941–943.

SONDERGAARD JO, RASMUSSEN MS, VIDEBAEK H, BERNSTEIN IT, MYRHOJ T, KRISTIANSEN VB, SOMMER P AND BULOW S. (1993). Mandibular osteomas in sporadic colorectal carcinoma. A genetic marker. *Scand. J. Gastroenterol.*, **28**, 23–24.

SPIRIO L, OLSHWANG S, GRODEN J, ROBERTSON M, SAMOWITZ, JOSLYN G, GELBERT L, THLIVERIS A, CARISON M, OTTERUD B, LYNCH H, WATSON P, LYNCH P, LAURENT-PUIG P, BURT R, HUGHES JP, THOMAS G, LEPPERT M AND WHITE R. (1993). Alleles of the APC gene: an attenuated form of familial polyposis. *Cell*, **75**, 951–957.

THIBODEAU SN, BREN G AND SCHAID D. (1993). Microsatellite instability in cancer of the proximal colon. *Science*, **260**, 816–819.

TRABOULSI EL, KRUSH AJ, GARDNER EJ, BOOKER SV, OFFERHAUS GJ, YARDLEY JH, HAMILTON SR, LUK GD, GIARDIELLO FM, WELSH SB, HUGHES JP AND MAUMENEE IH. (1987). Prevalence and importance of pigmented ocular fundus lesions in gardner's syndrome. *N. Engl. J. Med.*, **316**, 661–667.

UMAR A, BOYER JC, THOMAS D, NGUYEN DC, RISINGER JI, BOYD J, IONOV Y, PERUCHO M AND KUNKEL TA. (1994). Defective mismatch repair in extracts of colorectal and endometrial cancer cell lines exhibiting microsatellite instability. *Biol. Chem.*, **269**, 14367–14370.

UTSUNOMIYA J AND NAKAMURA T. (1975). The occult osteomatous changes in the mandible in patients with familial polyposis coli. *Br. J. Surg.*, **62**, 45–51.

VASEN HFA, MECKLIN J-P, MEERA-KHAN P AND LYNCH HT. (1991). The International Collaborative Group on Hereditary Non-Polyposis Colorectal Cancer (ICG-HNPCC). *Dis. Colon Rectum*, **34**, 424–425.

WALLIS YL, MCDONALD F, HULTEN M, MORTON JE, MCKEOWN CM, NEOPTOLEMOS JP, KEIGHLEY M AND MORTON DG. (1994). Genotype-phenotype correlation between position of constitutional APC gene mutation and CHRPE expression in familial adenomatous polyposis. *Hum. Genet.*, **94**, 543–548.

DE WIND N, DEKKER M, BERNS A, RADMAN M AND DE RIELE H. (1995). Inactivation of the mouse Msh2 gene results in mismatch repair deficiency, methylation tolerance, hyperrecombination, and predisposition to cancer. *Cell*, **82**, 321–330.